# *TP53* deficiency induces a low-adhesion transcriptomic signature correlating with accelerated CAR-T cell exhaustion in B-ALL

**DOI:** 10.3389/fimmu.2026.1842309

**Published:** 2026-05-28

**Authors:** Yu Wang, Yu-juan Xue, Yue-ping Jia, Ai-dong Lu, Le-ping Zhang, Pin-pin Sui, Hui-min Zeng

**Affiliations:** 1 Department of Pediatrics, Peking University People’s Hospital, Peking University, Beijing, China; 2 Molecular Diagnostic Center, Peking University People’s Hospital, Peking University, Beijing, China

**Keywords:** B-cell acute lymphoblastic leukemia, CAR-T cell therapy, ECM/adhesion pathway, immunological synapse, TP53 deficiency

## Abstract

**Background:**

Chimeric antigen receptor T-cell (CAR-T) therapy is a potent treatment for relapsed/refractory B-cell acute lymphoblastic leukemia (R/R B-ALL). However, patients harboring *TP53* alterations experience disproportionately high relapse rates, and the underlying tumor-intrinsic mechanisms driving this resistance remain poorly understood.

**Methods:**

We engineered isogenic *TP53*-wildtype and *TP53*-knockout NALM-6 B-ALL cell models using CRISPR/Cas9. The specific impact of p53 loss on CD19 CAR-T cell cytotoxicity, proliferation, and exhaustion was evaluated using *in vitro* co-culture assays. Associated molecular alterations and adaptive responses were profiled via RNA-sequencing (RNA-seq).

**Results:**

In *in vitro* assays, *TP53*-deficient B-ALL cells exhibited intrinsic resistance to CAR-T-mediated killing. Co-culturing with these *TP53*-null targets reduced CAR-T cell expansion, suppressed effector cytokine secretion, and accelerated a T-cell exhaustion phenotype, indicated by the co-expression of PD-1, TIM-3, and LAG-3. Transcriptomic profiling revealed that *TP53* deficiency induces a low-adhesion signature, characterized by the coordinated downregulation of core extracellular matrix (ECM) and cell adhesion genes, including *ITGB1* and *LAMA5*. This transcriptional profile suggests a structural remodeling that potentially deprives CAR-T cells of the mechanical anchoring requisite for establishing a stable immunological synapse (IS). Furthermore, *TP53*-deficient cells failed to activate immunogenic signaling pathways, such as IL-2/STAT5, under immune pressure.

**Conclusions:**

Our *in vitro* findings indicate that *TP53* deficiency in B-ALL cells downregulates adhesion networks and impairs immunogenic signaling, which correlates with accelerated CAR-T cell exhaustion. These transcriptomic and cellular observations suggest a potential link between *TP53*-mediated adhesion loss and CAR-T resistance, warranting further *in vivo* validation and biophysical investigations.

## 1 Introduction

Chimeric antigen receptor T-cell (CAR-T) therapy targeting CD19 has achieved remarkable success in relapsed/refractory B-cell acute lymphoblastic leukemia (R/R B-ALL). However, long-term durability remains a significant challenge, with a substantial fraction of patients eventually experiencing treatment failure (1–5). While antigen loss and poor T-cell persistence are well-recognized culprits (6–8), tumor-intrinsic genetic alterations that drive sophisticated immune evasion are increasingly coming to the forefront.

One such critical determinant is the tumor suppressor gene *TP53*. Although *TP53* deletions or mutations are relatively uncommon at initial ALL diagnosis, they are enriched in the relapsed setting and are strongly associated with adverse outcomes (9–12). In patients receiving CAR-T therapy, emerging retrospective clinical cohorts have consistently demonstrated that *TP53*-mutated cases exhibit significantly shortened event-free and overall survival, often presenting with atypical relapse patterns (13–16). These clinical observations underscore the possibility that loss of p53 function in leukemia cells directly diminishes their susceptibility to CAR-T cell eradication.

To explore this, we previously conducted transcriptomic profiling (RNA-seq) of B-ALL cells with varying *TP53* statuses. Interestingly, our bioinformatic analysis revealed a profound downregulation of genes encoding extracellular matrix (ECM) components and cell adhesion molecules. Given that these structural networks are increasingly recognized as important facilitators for the stabilization of the immunological synapse (IS) during T-cell and target cell interactions, we hypothesized that *TP53* deficiency might biophysically impair the quality of CAR-T engagement. In this study, we utilized CRISPR/Cas9-engineered isogenic NALM-6 cell lines and *in vitro* functional assays to systematically investigate how *TP53* deficiency impacts the susceptibility of B-ALL cells to CAR-T-mediated cytotoxicity. By integrating *in vitro* functional readouts with global transcriptomic profiling, we characterized a novel p53-dependent modulation of tumor adhesive properties and immunogenic signaling. Our findings suggest that *TP53* deficiency transcriptionally reprograms B-ALL cells into a low-adhesion state, which disrupts normal immunogenic responses (such as the IL-2/STAT5 signaling axis) and correlates with accelerated CAR-T cell exhaustion. This provides a novel cellular perspective on the structural mechanisms underlying CAR-T resistance, setting the stage for future *in vivo* studies to explore how this adhesion deficit interacts with the complex bone marrow tumor microenvironment.

## 2 Materials and methods

### 2.1 Cell lines and culture conditions

The human B-ALL cell line NALM-6 (ATCC^®^ CRL-3273™) was acquired from the American Type Culture Collection (ATCC, Manassas, VA, USA). All cells were cultured in RPMI-1640 medium supplemented with 10% fetal bovine serum (FBS; Gibco, Thermo Fisher Scientific, Waltham, MA, USA), 100 U/mL penicillin, and 100 μg/mL streptomycin (Gibco, Thermo Fisher Scientific). Cultures were maintained in a humidified incubator at 37 °C with 5% CO_2_. Routine testing for mycoplasma contamination was conducted using the MycoAlert Mycoplasma Detection Kit (Lonza, Basel, Switzerland) to ensure cell line integrity.

### 2.2 CRISPR/Cas9-mediated *TP53* gene editing

Isogenic NALM-6 cell lines with divergent *TP53* statuses (*TP53*^+/+^, *TP53*^–/–^, and *TP53* missense mutants) were generated using the CRISPR/Cas9 system. Briefly, single guide RNAs (sgRNAs) targeting the human *TP53* gene were cloned into the lentiCRISPR v2 vector (Addgene, Watertown, MA, USA). Lentiviral particles were packaged in HEK293T cells and subsequently utilized to transduce NALM-6 cells. Post-transduction, cells underwent puromycin selection, and single-cell clones were isolated via limiting dilution. The exact *TP53* knockout or missense mutations were validated by Sanger sequencing, and the complete absence of p53 protein expression in knockout clones was confirmed via Western blotting.

### 2.3 Isolation of primary T cells and generation of CD19 CAR-T cells

Primary human T cells were isolated from peripheral blood mononuclear cells (PBMCs) of two independent healthy donors using CD4 and CD8 MicroBeads (Miltenyi Biotec, Bergisch Gladbach, Germany), respectively. T cells were subsequently activated with CD3/CD28 Dynabeads (Gibco, Thermo Fisher Scientific) and cultured in X-VIVO 15 medium (Lonza, Walkersville, MD, USA) supplemented with 5% human AB serum and recombinant human IL-2 (100 IU/mL; PeproTech, Cranbury, NJ, USA). The percentages of CD4+ and CD8+ CAR-T cells were determined by flow cytometry, and the cells were formulated at a precise 1:1 ratio of CD4+ to CD8+ CAR-T cells for all subsequent functional and transcriptomic assays to ensure standardized effector potency and reproducibility. Following 48 hours of activation, T cells were transduced with a lentiviral vector encoding a second-generation CD19-specific CAR, which incorporates an anti-CD19 scFv, a CD8α hinge/transmembrane domain, a 4-1BB costimulatory domain, and a CD3ζ signaling domain. Transduction efficiency was evaluated 4–5 days post-transduction via flow cytometry, utilizing biotinylated Protein L (AcroBiosystems, Newark, DE, USA) followed by fluorophore-conjugated streptavidin (BioLegend, San Diego, CA, USA).

### 2.4 *In vitro* cytotoxicity and T-cell proliferation assays

To evaluate CAR-T cell-mediated cytotoxicity, *TP53*^+/+^, *TP53*^–/–^, or *TP53*-mutant NALM-6 target cells were co-cultured with CD19 CAR-T cells at various effector-to-target (E:T) ratios in 96-well plates (1:4, 1:8, 1:16, 1:32, and 1:64). A fixed E:T ratio of 1:16 was specifically selected for subsequent long-term functional and transcriptomic assays to provide a sub-maximal immune pressure, allowing for the observation of cumulative T-cell exhaustion and proliferation kinetics that might be obscured by rapid target clearance at higher ratios. After 48 hours of co-culture, the specific lysis of target cells was determined using flow cytometry by gating on the CD19^+^ target population. To assess long-term target cell survival and T-cell proliferation, absolute cell counts of both surviving CD19+ leukemia cells and CD3+ T cells were determined on day 0 and day 6 post-coculture using CountBright™ Absolute Counting Beads (Invitrogen, Thermo Fisher Scientific, Waltham, MA, USA). Untransduced T cells were utilized as a negative control, and cell apoptosis was evaluated using an Annexin V/7-AAD apoptosis kit (BD Biosciences, San Jose, CA, USA).

### 2.5 Cytokine release assay (ELISA)

The effector function of CAR-T cells was further assessed by quantifying cytokine production. CAR-T cells were co-cultured with the respective NALM-6 target cells at a 1:16 E:T ratio. Cell-free supernatants were collected at predefined time points (days 0, 2, 4, and 6) and stored at –80 °C until analysis. The concentrations of Granzyme B, Interferon-gamma (IFN-γ), and Tumor Necrosis Factor-alpha (TNF-α) were measured using commercially available Enzyme-Linked Immunosorbent Assay (ELISA) kits (R&D Systems, Minneapolis, MN, USA) according to the manufacturer’s instructions.

### 2.6 Flow cytometry and exhaustion phenotyping

To assess the exhaustion profile of CAR-T cells during prolonged antigen exposure, T cells were harvested from the co-culture system on day 7. Cells were washed and stained with a panel of fluorochrome-conjugated monoclonal antibodies against human CD3, CD4, CD8, and exhaustion markers including PD-1, TIM-3, and LAG-3 (all from BioLegend, San Diego, CA, USA). Data acquisition was executed on a BD LSRFortessa flow cytometer (BD Biosciences, San Jose, CA, USA). Final data analysis was analyzed using FlowJo software (Tree Star, Ashland, OR, USA).

### 2.7 RNA extraction, sequencing, and bioinformatics analysis

Total RNA was extracted from NALM-6 *TP53*^+/+^ and *TP53*^–/–^ cells (both at baseline and post-CAR-T co-culture) using TRIzol reagent (Invitrogen, Thermo Fisher Scientific). To distinguish baseline intrinsic gene expression from adaptive responses, RNA was collected both at baseline (untreated cells) and from residual CD19+ ALL cells sorted via flow cytometry on day 4 post-CAR-T co-culture. RNA integrity was verified using the Agilent 2100 Bioanalyzer. Library preparation and sequencing were performed on an Illumina NovaSeq 6000 platform (Illumina, San Diego, CA, USA) to generate 150 bp paired-end reads. Raw reads were mapped to the human reference genome (GRCh38) using HISAT2. Differential gene expression analysis was performed with the DESeq2 R package, with a significance threshold of |log_2_(Fold Change)| > 1 and an adjusted P-value < 0.05.

Gene Ontology (GO) enrichment analysis of differentially expressed genes was performed using the clusterProfiler R package to highlight biological processes. Gene Set Enrichment Analysis (GSEA) was utilized to evaluate the enrichment of immunological signatures (e.g., the HALLMARK_IL2_STAT5_SIGNALING pathway) using the MSigDB database.

### 2.8 Statistical analysis

Statistical analyzes were performed using GraphPad Prism software (version 9.0; GraphPad Software, San Diego, CA, USA). Quantitative data are presented as the mean ± standard deviation (SD). For *in vitro* functional assays (cytotoxicity, proliferation, and cytokine release), experiments were independently performed using CAR-T cells generated from two healthy donors, with at least three technical replicates per condition. Transcriptomic profiling and subsequent qRT-PCR validations were conducted using co-culture samples derived from one representative donor. Comparisons between two groups were analyzed using an unpaired Student’s t-test, whereas comparisons among multiple groups were evaluated using one-way or two-way analysis of variance (ANOVA) followed by appropriate *post hoc* tests. A P-value of < 0.05 was considered statistically significant.

## 3 Results

### 3.1 *TP53* deficiency confers intrinsic resistance to CAR-T cell-mediated cytotoxicity in B-ALL cells *in vitro*

**Figure 1:**
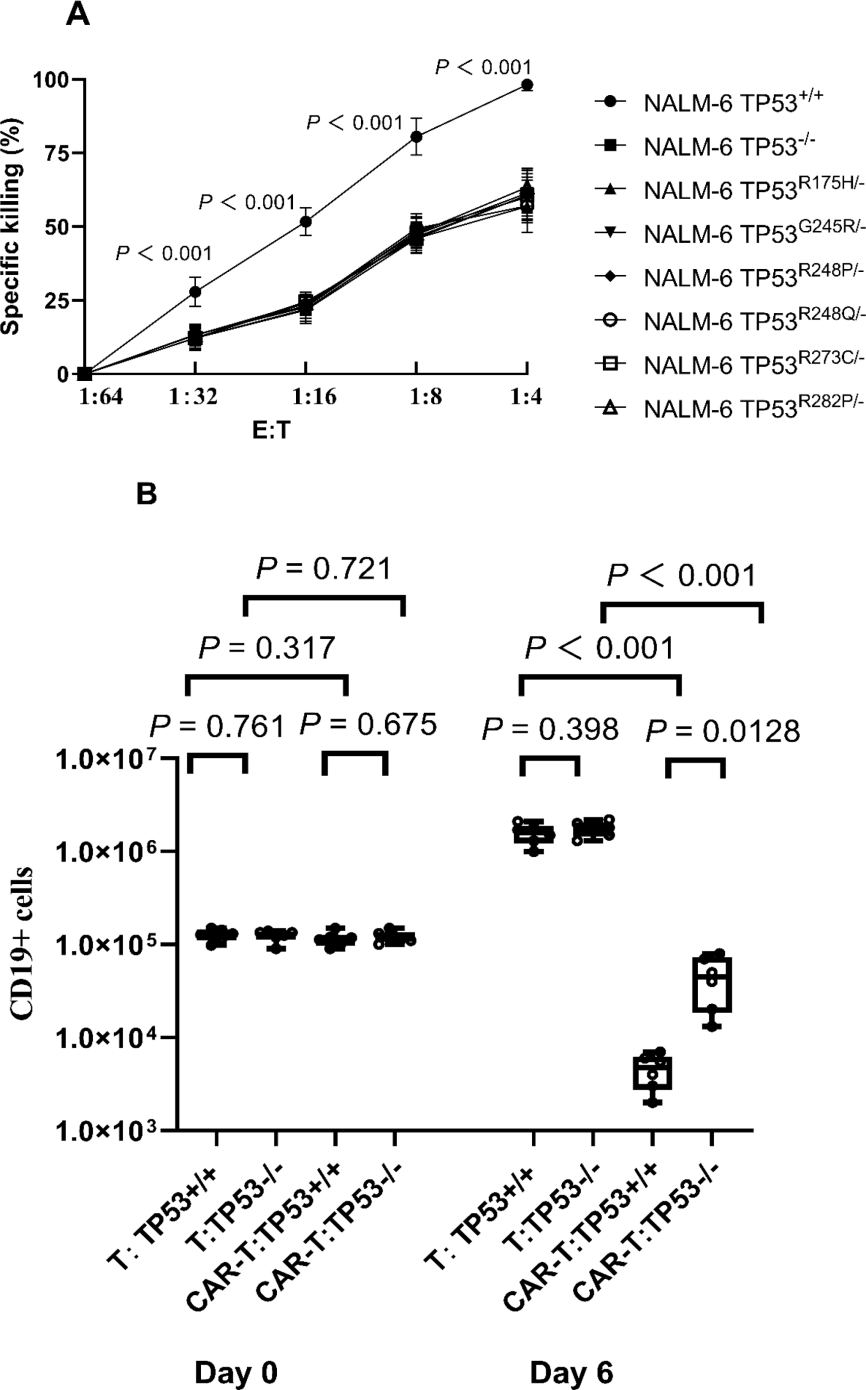
*TP53* deficiency confers intrinsic resistance to CAR-T cell-mediated cytotoxicity in B-ALL cells *in vitro*. **(A)** Specific lysis of NALM-6 cells harboring diverse *TP53* genetic statuses (wild-type *TP53*^+/+^, biallelic knockout *TP53*^-/-^, and multiple missense mutants including R175H, G245R, R248P, R248Q, R273C, and R282P) following a 48-hour co-culture with CD19 CAR-T cells at the indicated effector-to-target (E:T) ratios. **(B)** Absolute cell counts of surviving CD19+ target cells evaluated by flow cytometry on day 0 and day 6. NALM-6 *TP53*^+/+^ and *TP53*^-/-^ cells were co-cultured with either untransduced T cells (T) or CD19 CAR-T cells at a 1:16 E:T ratio. Statistical analysis: data are presented as the mean ± standard deviation (SD) from at least three independent experiments. For Panel **(A)**, statistical significance was determined using a two-way analysis of variance (ANOVA) followed by Dunnett’s multiple comparisons test, specifically comparing the *TP53*^+/+^ control group against all other *TP53*-deficient and missense mutant groups at each E:T ratio. Exact *P*-values indicating these specific comparisons are annotated in the image, with *P* < 0.05 considered statistically significant. Data are representative of independent experiments performed using CD19 CAR-T cells generated from two distinct healthy donors.

To determine whether the *TP53* gene status directly affects the sensitivity of B-ALL cells to CAR-T therapy, we evaluated CAR-T cytotoxicity against isogenic NALM-6 cell lines with distinct p53 functional states. Using CRISPR/Cas9 technology, we generated NALM-6 sub-lines carrying either wild-type *TP53* (NALM-6 TP53^+/+^), biallelic *TP53* knockout (NALM-6 *TP53*^–/–^), or common *TP53* missense mutations. All modified subclones expressed comparable levels of the CD19 target antigen [as confirmed by flow cytometric histograms and quantitative mean fluorescence intensity (MFI) statistics in **Supplementary Figures 1A, B**] and showed comparable *in vitro* growth characteristics, isolating *TP53* status as the primary variable. Upon co-culturing these ALL cells with CD19 CAR-T cells at varying effector-to-target (E:T) ratios (ranging from 1:64 to 1:4), we observed that *TP53* loss-of-function conferred a profound survival advantage to the leukemia cells *in vitro*. Both the *TP53*-knockout and *TP53*-mutant cells were significantly more resistant to CAR-T killing compared to the *TP53*-wildtype cells across all E:T ratios (**Figure 1A**). Furthermore, the absolute quantification of surviving target cells at a representative 1:16 E:T ratio confirmed this resistance, as *TP53*-deficient cells maintained a significant survival advantage over 6 days of continuous co-culture (**Figure 1B**). By contrast, control T cells lacking the CAR (untransduced T lymphocytes) did not induce appreciable leukemia cell death in any group, confirming that tumor clearance was dependent on CAR-mediated recognition of CD19. Because the *TP53* knockout and missense-mutant targets exhibited equivalent refractoriness, subsequent functional assays utilized the NALM-6 *TP53*^–/–^ cells as the representative model for complete p53 dysfunction. These data demonstrate that loss of normal p53 activity intrinsically blunts the susceptibility of ALL cells to CAR-T cell-mediated killing *in vitro*.

### 3.2 Interaction with *TP53*-deficient B-ALL targets imposes restricted proliferative capacity, attenuates effector fitness, and accelerates an exhaustion-associated phenotype in CAR-T cells

**Figure 2:**
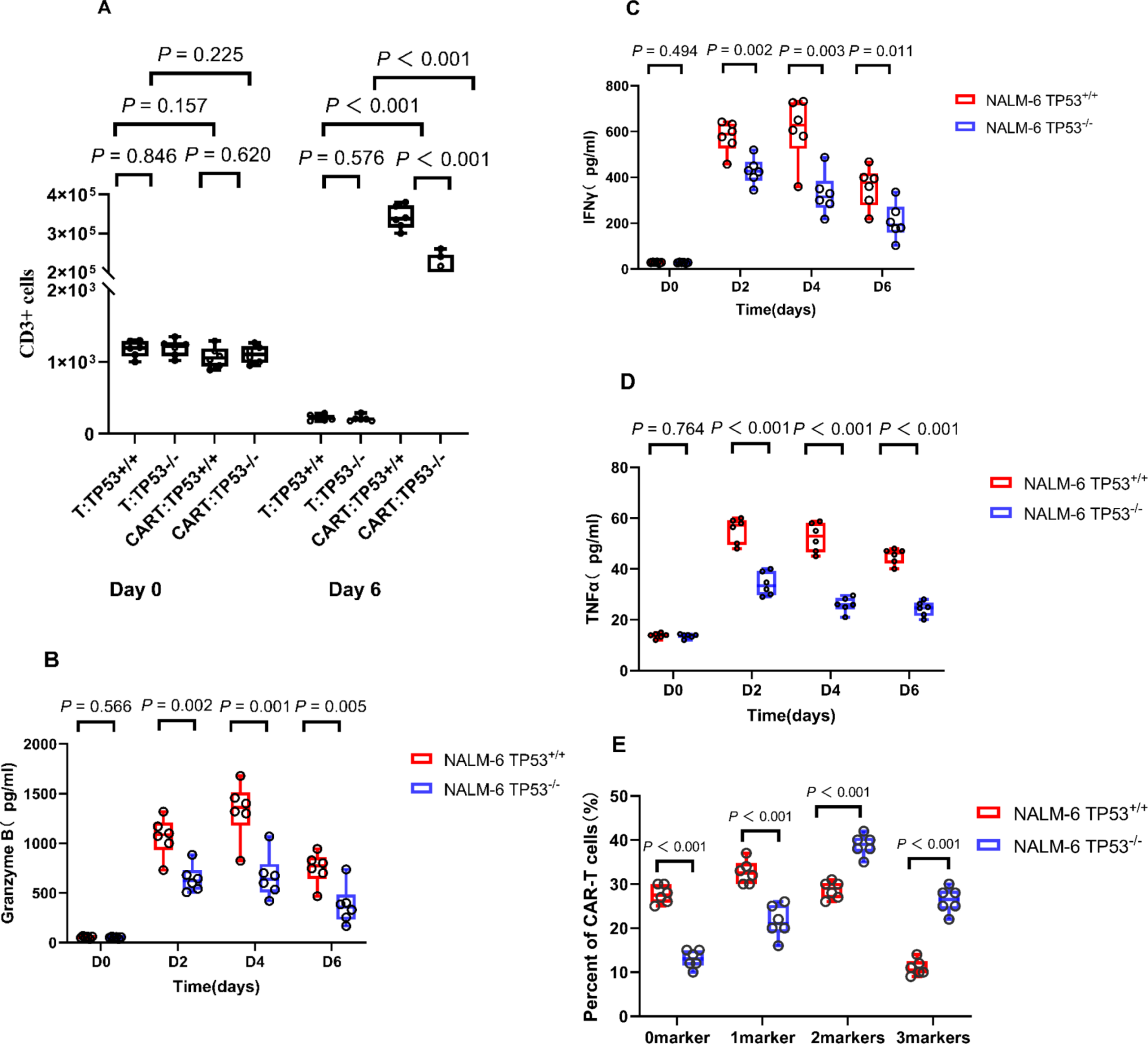
Interaction with *TP53*-deficient targets imposes restricted proliferative capacity, attenuates effector fitness, and accelerates an exhaustion-associated phenotype in CAR-T cells. **(A)** Absolute counts of CD3+ T cells (untransduced T cells or CAR-T cells) measured on day 0 and day 6 post-coculture with NALM-6 *TP53*^+/+^ or *TP53*^-/-^ target cells at a 1:16 E:T ratio, illustrating the restricted expansion of CAR-T cells facing TP53-deficient targets. **(B–D)** Quantitative analysis of effector cytokine secretion. The concentrations of granzyme B **(B)**, Interferon-gamma (IFN-γ) **(C)**, and tumor necrosis factor-alpha (TNF-α) **(D)** in the cell-free supernatants were measured by ELISA at predefined time points (days 0, 2, 4, and 6) during co-culture at a 1:16 E:T ratio. **(E)** The proportions of CAR-T cells co-expressing zero, one, two, or three inhibitory receptors (PD-1, TIM-3, and LAG-3) on day 7 of co-culture with NALM-6 *TP53*^-/-^ or *TP53*^+/+^ cells at a 1:16 E:T ratio. Statistical analysis: data are shown as the mean ± SD of a minimum of three independent experiments. Statistical significance among multiple groups was evaluated utilizing two-way ANOVA. Data are representative of independent experiments performed using CD19 CAR-T cells generated from two distinct healthy donors.

We next investigated how engaging *TP53*-deficient leukemia targets impacts the functional kinetics of CAR-T cells *in vitro*. By utilizing a fixed effector-to-target (E:T) ratio of 1:16 (selected as a stress-test model to allow for sustained interaction over 6 days), we observed that compared to co-cultures with NALM-6 *TP53*^+/+^ cells, CAR-T cells exposed to *TP53*^–/–^ cells exhibited a significantly restricted proliferative capacity. Over a 6-day co-culture, CAR-T cells exposed to *TP53*-wildtype targets underwent vigorous expansion (median ~4.1-fold increase), whereas CAR-T cells facing *TP53*-knockout targets showed significantly blunted proliferation (only ~2.3-fold expansion under the same conditions) (**Figure 2A**). This proliferation deficit was significantly pronounced by day 6 post-coculture, suggesting that p53-deficient leukemia cells fail to sustain optimal CAR-T cell expansion over time. This proliferation deficit was accompanied by a marked attenuation in effector cytokine output; the secretion levels of Granzyme B, IFN-γ, and TNF-α were consistently and significantly lower when CAR-T cells were challenged with *TP53*^–/–^ targets (**Figures 2B–D**). The consistently attenuated cytokine secretion in the *TP53*^–/–^ setting suggests that p53-deficient tumor cells provoke a weaker T-cell effector response. Furthermore, prolonged exposure to *TP53*-deficient ALL cells accelerated the acquisition of an exhaustion-associated phenotype. By day 7 of the co-culture, flow cytometric analysis revealed that the expression of key inhibitory receptors, including PD-1, TIM-3, and LAG-3, was significantly upregulated on the surface of CAR-T cells co-cultured with *TP53*^–/–^ cells compared to the wild-type controls (**Figure 2E**). However, this *TP53*-dependent skewing toward an exhausted T-cell phenotype was not observed in control cultures without CAR engagement. Collectively, these findings indicate that *TP53* abnormalities in tumor cells not only confer resistance to lysis but also actively induce a hypo-functional and exhausted state in the attacking CAR-T cells within the *in vitro* co-culture system.

### 3.3 Transcriptomic profiling reveals a TP53 deficiency-induced low-adhesion signature and defective immunogenic priming under CAR-T cell pressure

To uncover the tumor-intrinsic factors underlying the differential CAR-T responses, we performed comparative transcriptome analysis of *TP53*^+/+^ versus *TP53*^–/–^ NALM-6 cells. Bulk RNA sequencing was carried out on leukemia cells in two conditions: (a) untreated (baseline *TP53*-wildtype vs *TP53*-knockout ALL cells grown in standard culture), and (b) post-CAR-T co-culture (residual ALL cells sorted out after 4 days of interaction with CAR-T cells at a 1:16 E:T ratio, representing the transcriptional response of tumor cells under immune pressure). This design allowed us to distinguish baseline intrinsic gene expression differences due to *TP53* deficiency from differences in inducible gene programs during an immune attack.

**Figure 3:**
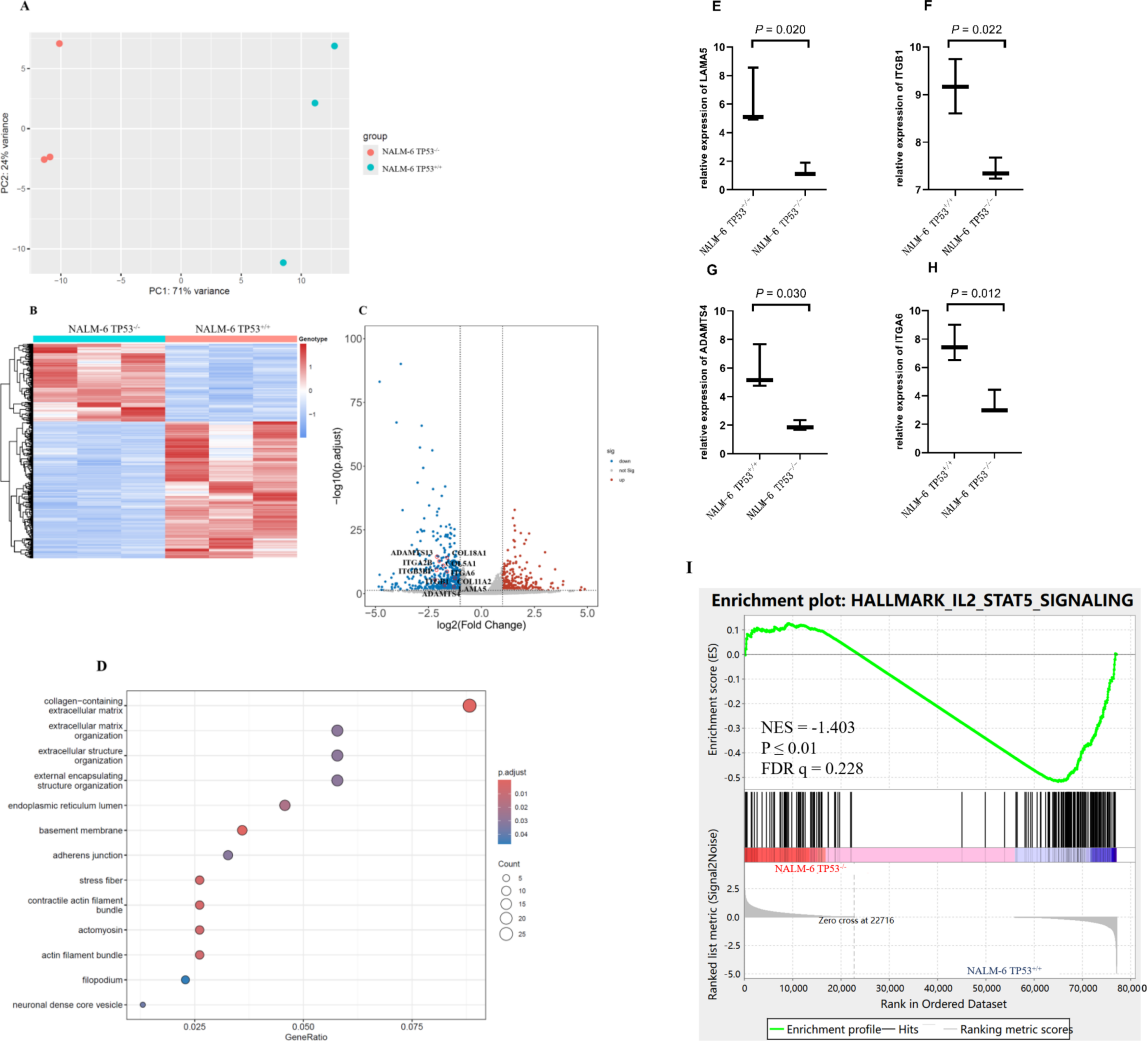
Transcriptomic profiling reveals a *TP53*-dependent low-adhesion signature and defective immunogenic priming under CAR-T cell pressure. **(A)** Unsupervised principal component analysis (PCA) demonstrating distinct global transcriptional clustering of NALM-6 *TP53*^+/+^ and *TP53*^-/-^ cells at baseline. **(B)** Heatmap illustrating the differentially expressed genes (DEGs) between *TP53*^+/+^ and *TP53*^-/-^ B-ALL cells, highlighting massive transcriptomic reprogramming. **(C)** Volcano plot emphasizing the coordinated downregulation of core extracellular matrix (ECM) and cell adhesion genes (e.g., LAMA5, COL5A1, ITGB1, ITGA6, ADAMTS4) in *TP53*-deficient cells. The significance threshold was set at |log_2_(fold change)| > 1 and adjusted *P* < 0.05. **(D)** Gene ontology (GO) enrichment analysis of the downregulated genes in *TP53*^-/-^ cells, revealing predominant enrichment in ECM structures, basement membrane, focal adhesions, and cytoskeletal components. **(E–H)** Validation of RNA-seq data via quantitative RT-PCR, confirming the significant downregulation of core adhesion-related genes: LAMA5 **(E)**, ITGB1 **(F)**, ADAMTS4 **(G)**, and ITGA6 **(H)** in *TP53*^-/-^ cells. **(I)** Gene set enrichment analysis (GSEA) evaluating the adaptive transcriptional response of residual ALL cells sorted after 4 days of CAR-T co-culture at a 1:16 E:T ratio, demonstrating significant suppression of the HALLMARK_IL2_STAT5_SIGNALING pathway in *TP53*^-/-^ cells under immune pressure (NES = -1.403, *P* ≤ 0.01, FDR q = 0.228). Statistical analysis: for qRT-PCR data **(E–H)**, comparisons between the two independent groups were conducted using an unpaired student’s t-test. Error bars represent the SD. Transcriptomic and qRT-PCR data represent three independent co-culture replicates utilizing CAR-T cells derived from one representative healthy donor.

Unsupervised principal component analysis (PCA) revealed distinct global transcriptional clustering based on *TP53* status (**Figure 3A**). Differential expression analysis identified a massive downregulation of gene networks in *TP53*^–/–^ cells, specifically enriched in ECM composition and cell adhesion functionalities. Volcano plots and heatmaps highlighted the coordinated suppression of key structural genes, including *LAMA5, COL5A1, COL18A1, ITGA6, ITGB1*, and *ADAMTS4* (**Figures 3B, C**). Crucially, GO enrichment analysis further revealed that the downregulated genes in TP53-deficient leukemia cells were predominantly localized to ECM structures, the basement membrane, focal adhesions, and cytoskeletal components, indicating compromised structural and adhesive integrity (**Figure 3D**). We validated the significant downregulation of core adhesion/ECM genes (*LAMA5, ITGB1, ADAMTS4, ITGA6*) via quantitative RT-PCR, mirroring the RNA-seq data (**Figures 3E–H**). This profound low-adhesion signature strongly implies a structural remodeling that may deprive CAR-T cells of the mechanical anchoring requisite to form a stable IS.

Finally, we utilized GSEA to evaluate the adaptive transcriptional response of residual ALL cells sorted after CAR-T co-culture. The analysis revealed that the IL-2/STAT5 signaling pathway was significantly suppressed in *TP53*^–/–^ cells relative to wild-type cells under immune pressure (NES = -1.403, *P* ≤ 0.01, FDR q = 0.228) (**Figure 3I**). *TP53*^+/+^ leukemia cells, when attacked by CAR-T cells, showed activation of an IL-2/STAT5 gene program, whereas *TP53*^–/–^ cells did not mount such a response. We interpret this as defective immunogenic priming in *TP53*^–/–^ leukemia cells under immunotherapeutic stress. *TP53*^–/–^ cells may be less able to sense or respond to the inflammatory and cytotoxic signals delivered by CAR-T cells. Importantly, to determine whether this transcriptomic suppression was merely a consequence of insufficient upstream IL-2 supply from the CAR-T cells or an intrinsic responsiveness defect within the tumor cells, we measured the IL-2 concentrations in the co-culture supernatants. ELISA revealed no significant difference in the secreted IL-2 levels between the *TP53*^+/+^ and *TP53*^-/-^ co-culture groups (**Supplementary Figure 2**). Given that the upstream paracrine IL-2 supply remained equivalent, the profound suppression of the IL-2/STAT5 signature in *TP53*^-/-^ targets rigorously confirms a severe impaired intrinsic responsiveness. These data suggest that *TP53* deficiency actively disrupts the signaling axis necessary for tumor cells to sense and respond to immune-derived stimuli, independently validating the functional failure at the immunological synapse. Together, these transcriptomic, bioinformatics, and cytokine analyzes suggest that *TP53* deficiency drives *in vitro* CAR-T resistance through a proposed dual mechanism: potentially compromising the stability of the CAR-T immune synapse via ECM/adhesion downregulation, and blunting the tumor’s internal immunogenic signaling response.

## 4 Discussion

In this study, we explored the tumor-intrinsic mechanisms underlying the compromised efficacy of CD19 CAR-T cells against *TP53*-deficient B-ALL *in vitro*. While previous studies have predominantly focused on how p53 loss disrupts downstream apoptotic execution (17), our integrated transcriptomic and functional profiling suggests an upstream, mechanobiological dimension to this resistance. We demonstrate that *TP53*-deficient leukemia cells exhibit a profound low-adhesion transcriptional signature, which temporally correlates with restricted CAR-T cell expansion, attenuated effector fitness, and the accelerated acquisition of an exhaustion-associated phenotype. Mechanistically, wild-type *TP53* is increasingly recognized as a master regulator of cellular adhesion and ECM homeostasis. In solid tumor models, p53 directly transactivates genes encoding laminins and integrins to maintain tissue integrity, whereas *TP53* loss triggers a disorganized, low-adhesion program characterized by the downregulation of core adhesion molecules (18, 19). Our transcriptomic findings in B-ALL perfectly mirror these solid tumor paradigms, revealing a broad suppression of the integrin/ECM-receptor network (e.g., *ITGB1, LAMA5, ADAMTS4*) upon *TP53* deletion. We hypothesize that this p53-dependent structural remodeling may serve as an intrinsic mechanism of immune evasion, creating a structural shedding that limits the mechanical anchoring available to CAR-T cells.

In the emerging field of physical immunology, stable cell-to-cell adhesion is recognized as a prerequisite for the formation and maintenance of a functional IS (20, 21). Integrin-mediated anchoring not only physically stabilizes the IS but also amplifies T-cell receptor (or CAR) signaling (22–25). We postulate that the structural destabilization of the tumor surface—inferred from the *TP53*-deficient transcriptome—deprives CAR-T cells of the mechanical tension required for optimal activation. Consequently, CAR-T cells may undergo frustrated or transient engagements with tumor targets. Continuous but sub-threshold synaptic signaling is a well-known driver of premature T-cell exhaustion, which perfectly aligns mechanistically with the rapid upregulation of PD-1, TIM-3, and LAG-3 observed in our *in vitro* co-culture system. Although direct biophysical visualization of the synapse is lacking in the current study, our comprehensive functional data—including blunted CAR-T proliferation, attenuated effector cytokine release, and rapid acquisition of an exhaustion-associated phenotype—strongly parallel the functional consequences of sub-optimal IS formation reported in the literature (24, 26).

This mechanobiological perspective strongly complements, rather than contradicts, existing downstream resistance mechanisms. For instance, Cox et al. recently demonstrated that *TP53* mutation impairs Fas/DR5 expression, conferring intrinsic resistance to CAR-T-induced apoptosis (27). Furthermore, our GSEA data revealed a defective immunogenic priming response, as *TP53*^–/–^ cells failed to upregulate the IL-2/STAT5 immunogenic signaling axis under immune pressure. Crucially, by confirming equivalent IL-2 concentrations in the co-culture microenvironment, we successfully decoupled upstream cytokine supply from downstream target responsiveness. This explicitly demonstrates that *TP53*^-/-^ leukemia cells harbor an intrinsic signaling paralysis, rendering them ‘deaf’ to paracrine inflammatory signals. It is highly plausible that the early upstream defect in IS structural coupling synergizes with these downstream signaling and apoptotic defects, cooperatively establishing a robust barrier to CAR-T efficacy (28). Recognizing this putative *TP53*-ECM-IS axis opens novel translational avenues. If defective adhesion acts as a structural barrier, therapeutic strategies aimed at remodeling the leukemia microenvironment could serve as potent sensitizers for CAR-T therapy (29, 30). For example, targeted agents such as Venetoclax, reported to upregulate specific integrins (e.g., *ITGB2*) in leukemia cells, might be repurposed as sensitizing probes to pharmacologically restore tumor adhesive properties prior to CAR-T infusion (31, 32). Alternatively, engineering next-generation CARs that provide enhanced integrin affinity might bypass the requirement for tumor-intrinsic adhesion.

Although our study establishes a novel mechanobiological framework linking *TP53* deficiency to CAR-T cell resistance, these foundational *in vitro* findings warrant further multi-dimensional exploration. Mechanistically, while we robustly captured an exhaustion-associated phenotype via surface inhibitory receptors and diminished cytokine secretion, future studies incorporating deeper epigenetic profiling, detailed kinetic analyzes of T-cell expansion, intracellular markers such as TOX, and functional reinvigoration assays are necessary to definitively define the reversibility of this dysfunctional state. Concurrently, direct biophysical visualization of the CAR-T IS using high-resolution live-cell imaging will be required to explicitly quantify the spatial synapse defects inferred from our transcriptomic signatures.

Ultimately, transitioning from our clean isogenic system to more heterogenous models is essential. Since the current model may not capture the full diversity of B-ALL, extending these findings to a broader range of cell lines, clinically relevant *TP53* missense mutants, and patient-derived primary cells will be critical to determine how the wider genetic background modulates this resistance. Furthermore, future *in vivo* studies utilizing patient-derived xenograft (PDX) models will be critical to validate the impact of this *TP53*-adhesion axis within the bone marrow niche. Exploring these avenues will further test the translational potential of adhesion-targeted combination therapies for this ultra-high-risk patient population.

## Data Availability

The datasets presented in this study can be found in online repositories. The names of the repository/repositories and accession number(s) can be found below: https://ngdc.cncb.ac.cn/gsa, GSA : HRA017411.
