# Supplementary material for: TP53 deficiency induces a low-adhesion transcriptomic signature correlating with accelerated CAR-T cell exhaustion in B-ALL
Source: Front Immunol. 2026 May 28;17:1842309. doi: 10.3389/fimmu.2026.1842309 (PMC13253516; doi:10.3389/fimmu.2026.1842309)
Supplement: Supplementary file 2 [file Table1.docx]

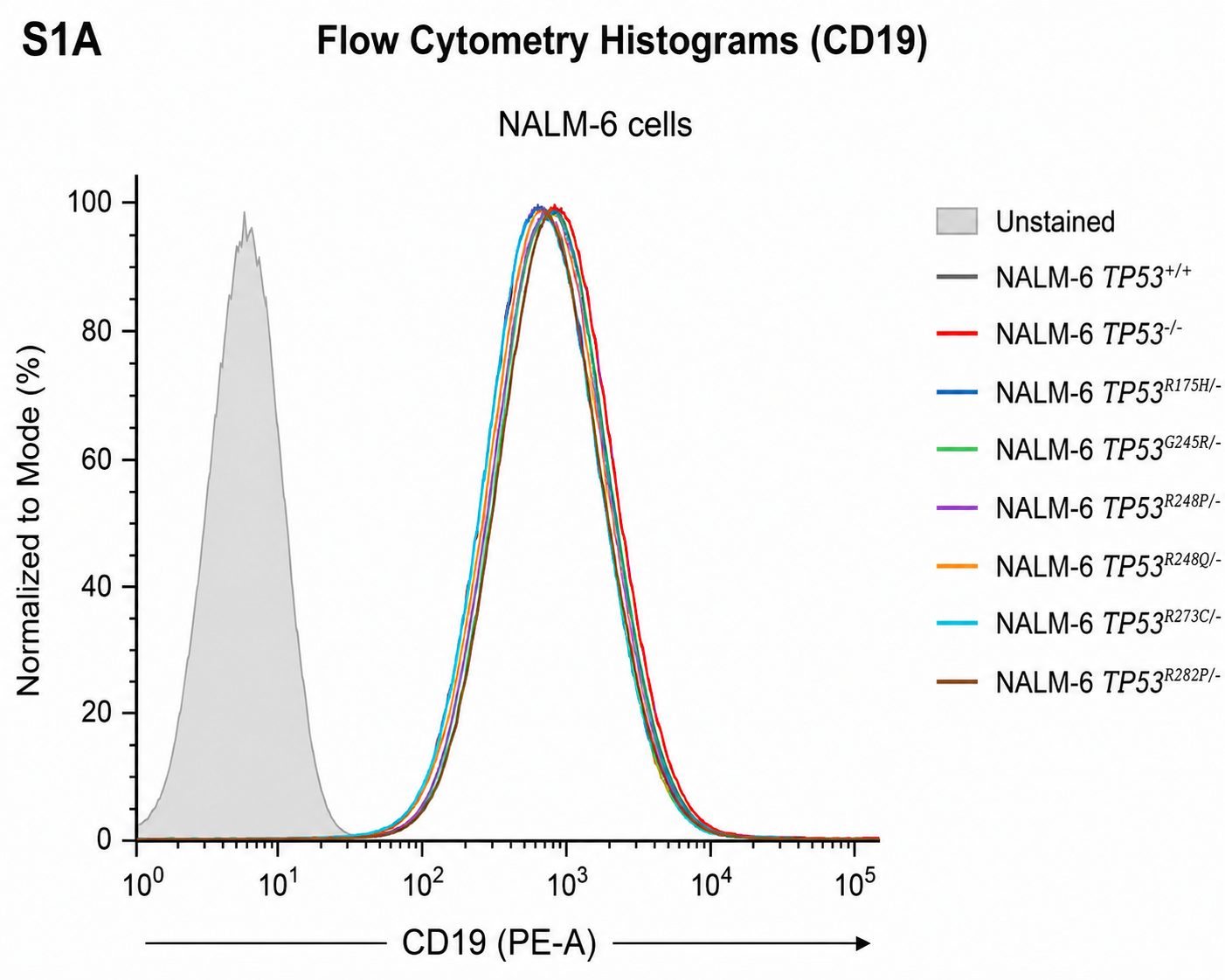


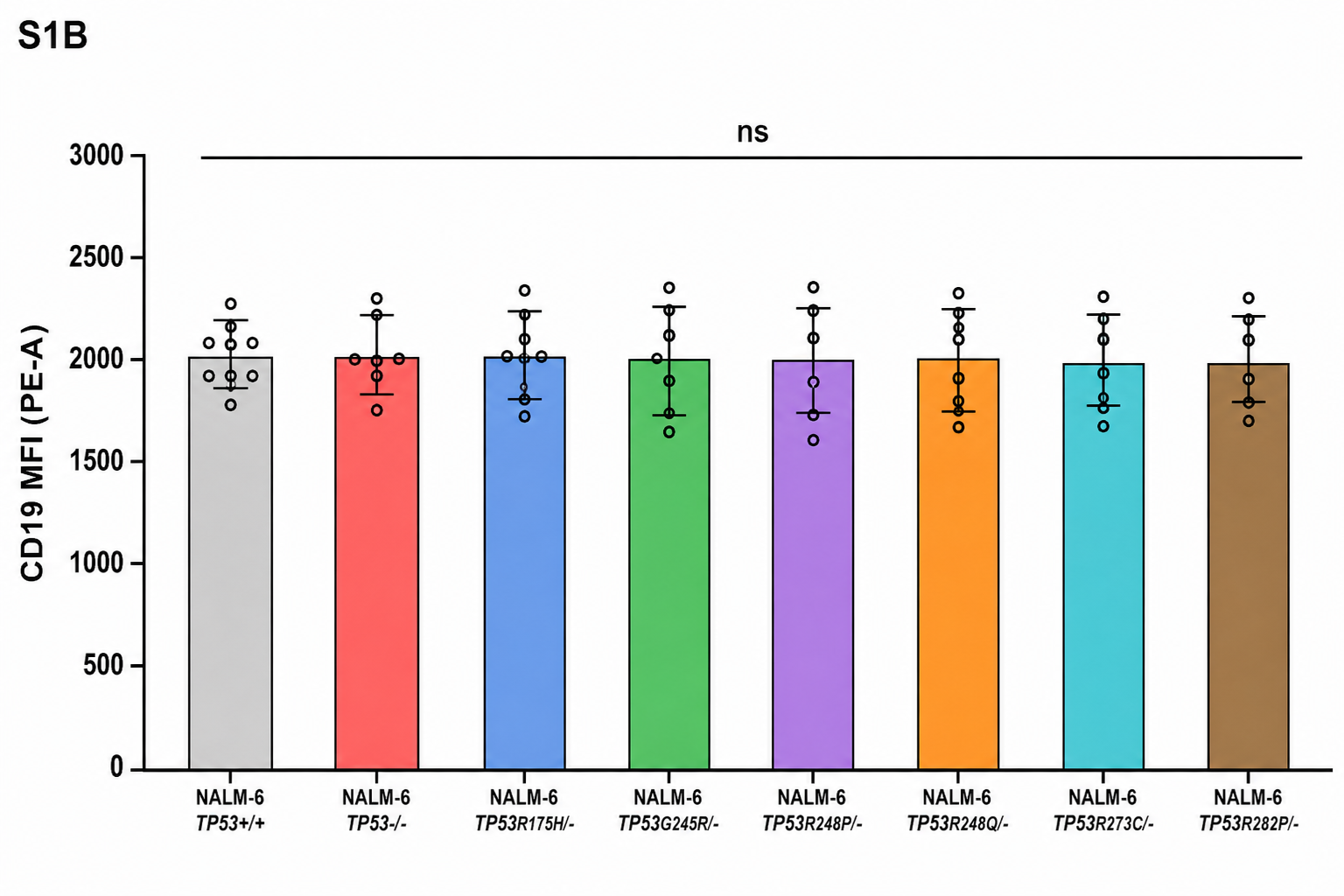


Supplementary Figure S1. Quality control of CD19 expression across *TP53*-modified NALM-6 subclones. (A) Representative flow cytometry histograms illustrating the overlay of CD19 expression on the cell surface of wild-type (*TP53*^+/+^), knockout (*TP53*^-/-^), and various missense mutant (*TP53*^R175H/-^, etc.) NALM-6 cells. The highly overlapping peaks indicate comparable antigen expression profiles across all established subclones. (B) Quantitative analysis of CD19 Mean Fluorescence Intensity (MFI). Data are presented as the mean ± SD of independent replicates. Statistical significance was determined using one-way ANOVA, confirming no significant difference (ns, P > 0.05) in CD19 expression levels among the different *TP53* genotypes, establishing *TP53* status as the sole biological variable.


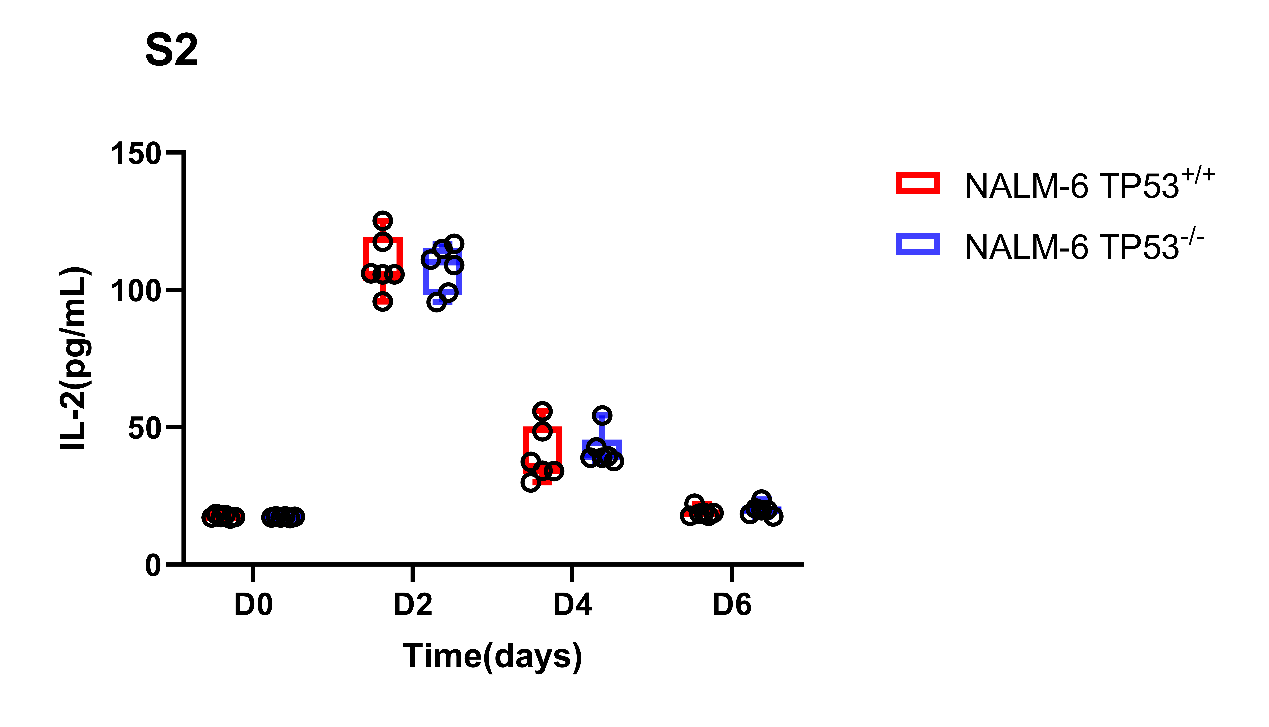


Supplementary Figure S2. IL-2 in the cell-free supernatants was measured by ELISA at predefined time points (days 0, 2, 4, and 6) during co-culture at a 1:16 E:T ratio.
